# Applying Mendelian randomization to appraise causality in relationships between smoking, depression and inflammation

**DOI:** 10.1038/s41598-022-19214-4

**Published:** 2022-09-03

**Authors:** D. Galan, B. I. Perry, V. Warrier, C. C. Davidson, O. Stupart, D. Easton, G. M. Khandaker, G. K. Murray

**Affiliations:** 1 Department of Public Health and Primary Care, University of Cambridge, Cambridge, UK; 2 Department of Psychiatry, University of Cambridge, Cambridge, UK; 3 Cambridgeshire and Peterborough NHS Foundation Trust, Cambridge, UK; 4 MRC Integrative Epidemiology Unit, Population Health Sciences, Bristol Medical School, University of Bristol, Bristol, UK; 5 Centre for Academic Mental Health, Population Health Sciences, Bristol Medical School, University of Bristol, Bristol, UK; 6 Avon and Wiltshire Mental Health Partnership NHS Trust, Bristol, UK; 7 Program in Complex Trait Genomics, Institute of Molecular Bioscience, University of Queensland, Brisbane, Australia

**Keywords:** Epidemiology, Depression, Risk factors, Genetic markers

## Abstract

Smoking, inflammation and depression commonly co-occur and may be mechanistically linked. However, key questions remain around the direction of association and the influence of residual confounding. We aimed to characterize the association between lifetime smoking and depression, as well as to assess the role that genetically-predicted C-reactive protein (CRP) level, (an archetypal generalized inflammatory marker) and/or IL-6 activity, as a potential explanation for this association. We performed inverse variance weighted Mendelian randomization (MR) analyses using recently published summary-level GWAS data for lifetime smoking index, CRP levels, and depression. A subset of inflammatory-related genetic variants from the lifetime smoking GWAS were also used to assess the potential inflammatory causal pathways between smoking and depression. The analysis indicated reciprocal relationships of lifetime smoking with depression (OR_Smk–Dep_ = 2.01, 95% CI 1.71–2.37, *p* < 0.001; OR_Dep–Smk_ = 1.09, 95% CI 1.06–1.13, *p* < 0.001), CRP levels and IL-6 activity (OR_Smk–CRP_ = 1.40, 95% CI 1.21–1.55, *p* < 0.001; OR_CRP–Smk_ = 1.03, 95% CI 1.02–1.05, *p* < 0.001, OR_IL-6/CRP–Smk_ = 1.06 (1.03–1.09), *p* < 0.001). These associations were also supported by the majority of the robust MR methods performed. We did not find evidence for a reciprocal relationship between CRP levels (using > 500 genetic instruments for CRP) and depression (OR_CRP–Dep_ = 1.01, 95% CI 0.99–1.04; OR_Dep–CRP_ = 1.03, 95% CI 0.99–1.07). We observed little variation in the IVW estimates between smoking and depression when we limited the genetic variants assessed to those related to measures of generalized inflammation, but we found evidence for an attenuation of the smoking-depression association in multivariable mendelian randomization when adjusting for IL-6 activity, suggesting that the IL-6 pathway may be at least in part responsible for the association of smoking and depression. Our study supports potential bidirectional causal associations between lifetime smoking and depression which may be at least in part explained by the IL-6 signalling pathway. The IL-6 pathway may represent a putative therapeutic target for smoking and to mitigate the effects of smoking on depression.

## Introduction

Increasing evidence indicates a role for inflammation, the tissue response to potentially harmful stimuli, in the pathogenesis of mental health disorders, particularly depression^1,2^. Even though, historically, the central nervous system (CNS) has been considered an immuno-privileged region in the human body, research has shown that microglia in the CNS produce inflammatory cytokines and inflammatory processes outside of the CNS can result inflammatory responses within the CNS^3–8^. Furthermore, blood levels of peripheral markers of inflammation, such as C-reactive protein (CRP) and interleukin IL6^2^, are elevated in depression in case–control studies and meta-analysis, and diseases associated with inflammation, such as rheumatoid arthritis, diabetes mellitus, coronary heart disease, stroke have been associated with depression^9,10^.

Modifiable exposures, such as smoking behavior, have been associated with both depression and inflammation. A 2014 study in the US estimated that the smoking prevalence among participants 16% and 40% in patients without a psychiatric and those who had a diagnosis of MDD during the previous year, respectively^11^. Furthermore, multiple lines of evidence indicate that smoking has pro-inflammatory molecular effects^12^, and observational studies support a systemic elevation of serum inflammatory markers in smokers^12^, including increases in CRP levels^13–15^. However, the evidence regarding direction of association and causality is unclear due to the biases inherent to observational studies^16,17^.

Mendelian randomization (MR) is an epidemiological approach that uses genetic variants as instruments to untangle the problems of reverse causation (genetic variants are fixed at conception; hence, genetically predicted levels of risk factors must precede any event) and unmeasured confounding (genetic variants show considerably less conventional confounding than phenotypic variables)^18^. If genetically-predicted values of a risk factor are associated with a specific disease outcome, then it is likely that the association between the risk factor and outcome has a causal basis^19–22^. In this manner, MR studies act like natural randomized control trials and overcome some of the biases of observational studies^23^.

Previous studies have shown through MR analyses that smoking and depression may have a bidirectional causal relationship^24^, although the mechanism through which smoking causes depression is not known. Given phenotypic associations between smoking and inflammation, and inflammation and depression, a plausible pathway from smoking to depression is via smoking’s pro-inflammatory effects. Whilst multiple cytokines are involved in inflammation, the combination of the strength of the evidence for phenotypic associations between CRP and depression, and the fact that GWAS for CRP are much larger than for any other molecular inflammatory biomarker, provide motivation for using CRP as a proxy for inflammation in the investigation of the aetiology of depression. Although previous MR studies using CRP as a proxy for systemic inflammation have shown conflicting evidence regarding the association between higher CRP levels and risk for depression^20,25,26^, recently, larger and better powered genome wide association studies (GWAS) for depression^27^ and for CRP^28^ have become available, and provide an opportunity to use improved genetic instruments, which explain a larger proportion of the variance, for causal inference to resolve prior ambiguities. Furthermore, CRP is a downstream marker of the interleukin (IL)-6 pathway , with IL-6 stimulating the release of CRP from hepatocytes^29^. The IL-6 pathway has been implicated in the pathogenesis of depression^30–32^, and smoking may lead to increased activity of the IL-6 signalling pathway^33^. The IL-6 pathway is therefore a hypothetical mechanism linking smoking behavior with depression.

In this study, the first to examine potential causality and direction of association, we conducted univariable and bidirectional MR analysis testing associations of genetically-predicted smoking behavior with CRP levels (as a measure of generalized inflammation), IL-6 activity, and risk of depression, and vice versa. Second, to examine for a potential mediating role of inflammation between smoking behavior and risk of depression, we conducted univariable MR analyses limiting the smoking exposure genetic variants to those which have been previously associated with inflammatory traits. Finally, we conducted a multivariable Mendelian randomization (MVMR) analysis to examine the associations between genetically-predicted smoking behavior and risk of depression after adjusting for genetically-predicted proxies of generalized inflammation (CRP) and IL-6 activity. We used the latest GWAS data to develop statistically better powered genetic instruments compared to previous studies and used this to investigate if inflammation mediates the potential causal effect of smoking on depression.

## Methods

All methods were performed in accordance with the relevant guidelines and regulations.

### Data sources

This study used *publicly available summary level data* obtained from previously published genome-wide association studies (GWAS) for all analyses. The source and description of the GWAS summary statistics used in this report for lifetime smoking, depression, and C-reactive protein (CRP) are presented in Table 1.

**Table 1:** Characteristics of the GWAS from which the summary statistics were obtained.

Variable	GWAS Source	Population Used	Total Participants	Cases	Controls	SNPs Covered	Genome-wide significant SNPs
Lifetime Smoking Index	Wootton et al^24^	UK Biobank	462,690*	NA	NA	7,683,352	126
Depression	Howard et al. /PGC^27^	UK Biobank & 33 cohorts	500,199	170,756	329,443	8,483,301	50
C-Reactive Protein Levels	Han et al^28^	UK Biobank	418,642	NA	NA	8,927,092	526

NA: Not applicable as continuous variable.

*249 318 never smokers, 164 649 former smokers and 48 723 current smokers.

#### Lifetime smoking index

Lifetime smoking index was selected as the variable to represent the smoking exposure for all analyses. Wootton et al. generated this lifetime smoking index, encompassing information regarding smoking heaviness, duration, and smoking initiation and cessation. One standard deviation increase in lifetime smoking score is equivalent to an individual smoking 20 cigarettes a day for 15 years and stopping 17 years ago or an individual smoking 60 cigarettes a day for 13 years and stopping 22 years ago^24^.

#### Depression

Depression summary statistics were obtained from the Psychiatric Genetic Consortium (PGC), as described by Howard and colleagues^27^, with 23andMe data excluded as full summary statistics with 23andMe data were not publicly available. The total number of contributing individuals is 500,199 (170,756 cases and 329,443 controls) with 8,483,301 variants analysed. (See Supplementary Table 1 for details of depression definitions in contributing cohorts).

#### Generalized inflammation (C-reactive Protein)

C-reactive protein (CRP) was selected as a downstream proxy for systemic inflammation^6,14,15,34,35^. Han et al. (2019) generated the summary statistics used for this study from 418,642 individuals of British ancestry in the UK biobank for whom CRP levels were available and whose levels were lower than 10 mg/L^28^.

#### IL-6 activity

Since CRP is a relatively generalized and downstream inflammatory marker, we also included an instrument for the genetically-predicted effects of IL-6 signalling on CRP levels, as a measure of *IL-6 activity*. This instrument was obtained from Georgakis et al^36^, and is derived of the effect of SNPs in the *IL6R* gene region on circulating CRP levels, derived from a large-scale GWAS of 200,402 European participants^37^.

### Selection of genetic instrumental variable

In Mendelian randomization, for a genetic variant to be a valid instrumental variable (IV), it must meet three assumptions: (i) the variant is associated with the exposure, (ii) the variant is not associated with any confounder of the exposure-outcome association, (iii) the variant does not affect the outcome, except via its association with the exposure^18^. The process for IV selection from the GWAS summary statistics for each of the variables studied was performed following a primarily statistical approach^38^. However, in some cases, the IV selection was further refined to include biological factors, as described below.

#### Statistical selection

SNPs were considered significantly associated to the GWAS variable of interest if the GWAS p-value reported on the summary statistics was smaller than 5 × 10^−8^^39^. Using multiple correlated variants representing the same effect would decrease the efficiency of the analyses and increase the risk of weak instrument bias in the estimates obtained without increasing the power of the study^40,41^. Consequently, absence of linkage disequilibrium (LD) and independence of the final IVs selected was ascertained using the *ld_clump()* function from the *ieugwasr* R package (clumping window kb = 10,000, *r*^*2*^ = 0.001)^42^.

If an IV selected did not have a match in the outcome GWAS statistics, a proxy IV (in linkage disequilibrium with the original IV; *r*^*2*^ > 0.8), was used instead. Proxy IVs were obtained using the *LDlink* R package^43^.

#### Biological selection

##### Smoking inflammatory SNPs

Inflammatory traits were defined as those relating to cytokines, acute phase proteins, and immune cells. Out of the lifetime smoking IVs from the Wooton et al. GWAS^24^ determined significant and in linkage disequilibrium, those that had been associated with inflammatory traits (*p* < 5 × 10^−8^) in previously published GWAS were subselected using the *Phenoscanner* R package^44^. These inflammatory-related IVs were used to further assess the role of inflammation in the association between smoking and depression.

##### Generalized inflammation (CRP) cis SNPs

When assessing the role of CRP, four variants—rs1205, rs3093077, rs1130864 and rs1800947—were selected as cis-variants, which are those located in the CRP gene region^20^. Limiting the analysis to cis-variants allows for more reliable conclusions due to the biological relevance of the variants used^38^. Clumping was performed using the *ld_clump()* function from the *ieugwasr* R package (clumping window kb = 10,000, *r*^*2*^ = 0.001, *p* = 0.99)^42^ and the variant rs3093077 was selected as the lead CRP variant. Wald ratio MR analyses^38^ were performed to assess the effect of this variant on depression and smoking.

##### IL-6 Activity cis SNPs

As per Georgakis *et al*^36^, we used seven variants (rs73026617, rs12083537, rs4556348, rs2228145, rs11264224, rs12059682, rs3469607) which are located within the *IL6R* coding region. Georgakis and colleagues coded their data to reflect associations of these variants with increased circulating CRP levels. We used the same coding for our analysis.

#### Univariable reciprocal Mendelian randomization analysis

**Figure 1:**
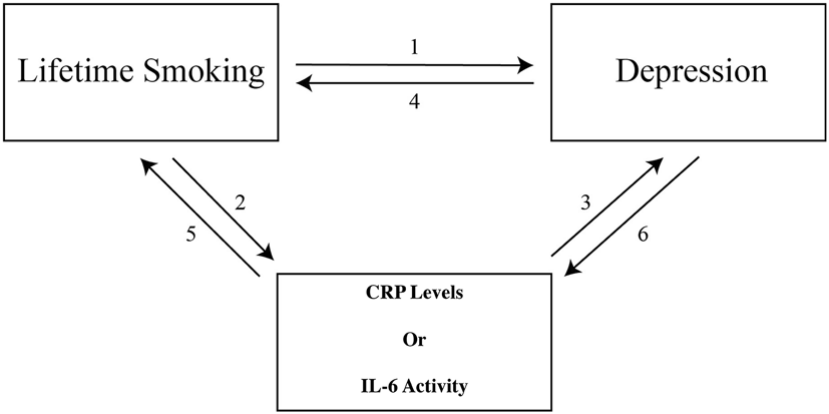
Graphic representation of the different univariable MR analyses performed. The. numbers for the different analyses correspond to those listed in Table 2.

The Inverse-variance weighted method was selected as the main method to calculate the combined effect of the selected instrumental variables in all univariable MR analyses. The IVW method is similar to a weighted regression of the effect of each specific IV on the outcome on the effect of the same IV on the exposure, restricting the intercept to zero^45,46^. All of the univariable MR analyses are represented in Fig. 1 and in Table 3. The direction of the relationships was confirmed using Steiger filtering using the *steiger_filtering()* function from the *TwoSampleMR* R package^47,48^. Steiger filtering infers the direction of the effect between two variables for each specific IV by assessing the size of the effect of the IV on each variable and its measurement error^47^.

##### Heterogeneity and sensitivity analysis

For all of the analyses performed, F-statistics were calculated to assess the strength of the instruments^49^. The heterogeneity between the estimates of the different IVs used was assessed using leave-one-out analyses, and visualizing the data with funnel, scatter, and forest plots^48,50,51^. The Funnel plots allow for the assessment of unbalanced directional pleiotropy; asymmetric distribution of the variants around the estimate would suggest unbalanced directional pleiotropy and could lead to bias in the results^51^. The scatterplot includes the SNP effect on the exposure on the x-axis versus the SNP effect on the outcome on the y-axis for every IV. Moreover, linear regression slopes represent the overall estimates of the effect on the exposure on the outcome. These plots highlight the heterogeneity of the Wald ratio estimates^51^. If all variants are valid IVs, a dose–response relationship should be observed in the scatterplot. If the majority of variants are valid IVs, pleiotropic variants may be detected as outliers in this plot, which would potentially require further investigation into the validity of those IVs based on the IV assumptions. Similarly, forest plots of the Wald ratios of every IV used along the overall IVW estimate allow for the detection of outliers.

The robustness of the overall estimate obtained with the IVW method was assessed using a combination of robust MR methods from different classes and working under a wide range of assumptions. Four additional MR methods were used: weighted median, MR-PRESSO, MR-Egger, and contamination mixture. Weighted median is a median based estimate that provides a valid estimate assuming 50% of the weight is originated from valid instruments. MR-PRESSO^52^ accounts for pleiotropy by detecting outliers through the comparison of the residual sum of squares of all the IVs with the expected distance under the null hypothesis of no horizontal pleiotropy. The MR-PRESSO results in this study represent the IVW analysis of the remaining IVs after removing the detected outliers.

MR-Egger accounts for pleiotropy by including an intercept term in the IVW model^53^. MR-Egger is severely biased by violations of the assumption that the variance of the individual IV effect estimates is negligible^53^. The *I*^*2*^*GX* is an adaptation of the *I*^*2*^ statistic from meta-analysis, related to the degree of dilution of the causal effect estimate; it ranges between 0 and 1 and values closer to 1 indicate less dilution of the causal effect estimate^51^. Another useful statistic in MR-Egger analyses is *Q*_*R*_, which compares the relative fit of the MR-Egger and IVW models to the estimates of every genetic variant used. When MR-Egger and IVW estimates differ and *Q*_*R*_ is close to 1, the IVW estimate is preferred, whereas when *Q*_*R*_ is close to zero, the MR-Egger estimate is preferred^54^.

Contamination mixture accounts for heterogeneity in causal mechanisms by identifying genetic instruments with similar causal estimates^55^. Under the assumption that out of all the different values taken by ratio estimates in large samples, the true causal effect is the value taken for the largest number of genetic variants, the contamination mixture method is robust even when more than 50% of the instruments are invalid^55^.

Although clumping reduces bias due to LD, to address the risk that SNPs located in the same region may be residual in LD with each other and so may bias results by 'double-counting' of effects, we used correlation-corrected IVW as a further sensitivity analysis. Correlations between the variants were obtained from a European sample from the 1000 genomes dataset. LD matrices were obtained using the TwoSampleMR package^48^, and analysis performed using the MendelianRandomization R package^56^.

#### Multivariable Mendelian randomization analysis

Multivariable MR (MVMR) methods allow for the estimation of the proportion of the effect of smoking directly acting on depression and the proportion potentially being explained by either CRP levels (as a measure of generalized inflammation) or IL-6 activity specifically. As for the univariate analysis, we used the 2SampleMR R package for the multivariable analysis. We selected instuments at the same genome-wide threshold of significance for each variable, clumped them, combined them, and clumped again, using the same clumping window as for the univariate analysis. We also conducted a sensitivity analysis using the MendelianRandomization R package^56^, using correlation correction.

## Results

### Instrumental variable selection

The results from the IV selection process, including all of the significant SNPs in each GWAS and the final IVs selected for each variable are shown in Supplemental Table 2.

**Table 2:** Univariable MR analyses performed in this report. Analyses 1, 2, and 3 are the main analyses in this report and the rest are considered supplemental. Analyses 4, 5, and 6 are the reciprocal MR analysis for 1, 2, and 3, respectively. Analyses 1b, 1c, and 3b include biological factors in the IV selection. Analyses 3b and 5b correspond to those performed using IL-6 activity.

Analysis	Exposure	Outcome
1	Lifetime Smoking	Depression
1b	Lifetime Smoking – Inflammation	Depression
1c	Lifetime Smoking – non-Inflammation	Depression
2	Lifetime Smoking	CRP
3	CRP	Depression
3b	IL-6 Activity	Depression
4	Depression	Lifetime Smoking
5	CRP	Smoking
5b	IL-6 Activity	Smoking
6	Depression	CRP

### IVW MR Analyses testing association of smoking with depression and CRP

The IVW method supported evidence for associations of genetically-predicted lifetime smoking index with risk of depression (ORSmk–Dep = 2.01, 95% CI : 1.71–2.37, *p* < 0.001), and CRP levels (ORSmk–CRP = 1.40, 95% CI: 1.27 − 1.55, *p* < 0.001). The IVW method also identified evidence for associations of genetically-predicted depression and lifetime smoking index (ORDep-Smk = 1.09, 95% CI: 1.06–1.13, *p* < 0.001), and between genetically-predicted CRP levels and lifetime smoking index (ORCRP–Smk = 1.03, 95% CI: 1.02–1.05, *p* < 0.001) (Supplementary Table 3). However, we did not find strong evidence for causal associations of CRP with depression.

### MR Analysis using inflammation-related genetic variants for smoking as IVs

Further analysis using the smoking-related genetic variants that are also associated with inflammation as IVs, the evidence for associations of genetically predicted smoking and depression remained for both inflammation-related and unrelated genetic variant sets (Supplementary Table 3).

### MR Analysis using cis variants for CRP and IL-6 activity

No association was observed between the lead CRP-cis variant, rs3093077, and depression or smoking. However, there was evidence for an association of a genetically-predicted effect of IL-6 activity on CRP levels with smoking (OR_IL-6/CRP–Smk_ = 1.06 (1.03–1.09), *p* < 0.001, but not depression (OR_IL–6/CRP-Dep_ = 0.93 (0.85–1.02), *p* = 0.126 (Table 3 and Supplementary Table 3).

**Table 3:** Inverse Variance Weighted Estimates for the univariable MR analyses.

Exposure	Outcome	SNPs	OR	Lower CI	Upper CI	StdErr	p-val
Smoking	Depression	126	2.01	1.71	2.37	0.08	7.78E−17
Smoking (Inflammatory)	Depression	15	1.89	1.23	2.91	0.22	0.004
Smoking (nonInflammatory)	Depression	114	2.07	1.73	2.47	0.09	6.97E−16
Smoking	CRP	126	1.4	1.27	1.55	0.05	2.89E−11
CRP	Depression	512	1.01	0.99	1.04	0.01	0.225
CRP (cis)*	Depression	1	1.00	0.92	1.09	0.04	1.000
IL-6 Activity	Depression	7	0.93	0.85	1.02	0.05	0.127
Depression	CRP	50	1.03	0.99	1.07	0.02	0.110
CRP	Smoking	521	1.03	1.02	1.05	0.01	1.71E−10
CRP (cis)*	Smoking	1	0.98	0.95	1.01	0.01	0.171
IL-6 Activity	Smoking	7	1.06	1.03	1.09	0.03	6.36E−05
Depression	Smoking	50	1.09	1.06	1.13	0.02	3.15E−07

Abbreviations: Smoking (Inflammatory) indicates smoking IVs previously associated with inflammation. Smoking (non Inflammatory) indicates smoking IVs not previously associated with inflammation. “Low CI” lower limit of 95% confidence interval. “Up CI” upper limit of 95% confidence interval.’

*Wald ratios were used to obtain the estimate for the CRP (cis) variant.

#### Visual assessment of MR estimates

**Figure 2:**
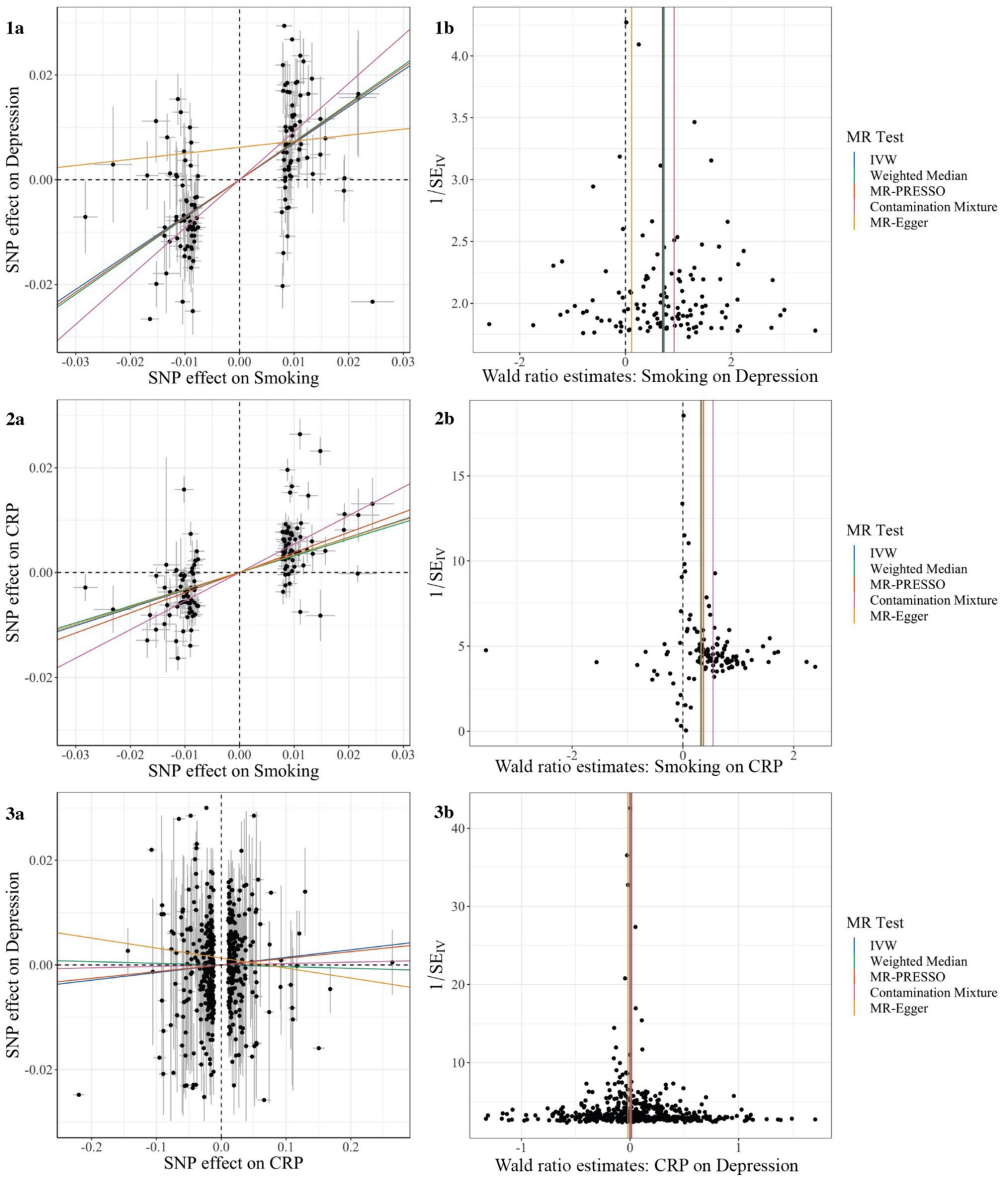
Main analyses: Effect of (**1**) smoking on depression, (**2**) smoking on CRP, and (**3**) CRP on depression. (**a**) Scatterplot showing the relationship between the variant-depression associations (x-axis) and the variant-smoking associations (y-axis) with standard error bars. The slopes of the colored lines correspond to the estimated causal effect obtained with each method used. (**b**) Funnel plot showing the relationship between the causal effect of the exposure on the outcome estimated using the Wald ratio estimate for each IV (x-axis) against the inverse of the standard error of the such estimate (y-axis). Vertical lines show the causal estimates using all SNPs combined into a single instrument for each of five different methods.

Visual assessment of the individual IV estimates using scatter, funnel, and forest plots indicated the presence of moderate heterogeneity among these effect estimates (Fig. 2 and Supplemental Figs. 1, 2, 3). However, the symmetrical distribution of individual estimates around the overall estimates suggests that the pleiotropy present is likely balanced.

### Results for sensitivity analyses

#### Influential IVs

We did not find evidence for single influential SNPs that may have biased results in leave-one-out tests for all analyses (Supplementary Fig. 2).

#### Results using robust methods

In the smoking-CRP, smoking-IL-6 activity, smoking-depression, and depression-smoking analyses, all of the global estimates calculated using robust MR methods, excluding the MR-Egger estimate, showed a similar increase in the odds of the outcome and higher levels of the exposure. I^2^GX values calculated indicate that the MR-Egger was likely to have provided biased estimates for analyses using smoking as an exposure; furthermore, the QR values obtained did not support a better fit of the MR-Egger data to the model when compared to the IVW model (Supplementary Table 5). Although the IVW model did not demonstrate an effect of depression on CRP, MR-PRESSO and contamination mixture analysis did demonstrate this effect (Supplementary Table 3).

### Multivariable MR

The estimated effect of smoking on depression remained significant after adjusting for the effect of CRP (OR = 2.00; 95% CI : 1.61–2.48; *p* = 3.5 * 10^–8^). When CRP was assessed as a mediator, it did not have a significant effect on depression (OR = 0.98; 95% CI : 0.92–1.04; *p* = 0.48) (Supplementary Table 4). However, when including the instrument for the effect of IL-6 activity with lifetime smoking in MVMR analysis, estimates for both smoking (IVW OR = 0.94 (95% CI: 0.76–1.17, *p* = 0.602)) and IL-6 activity (IVW OR = 1.03 (95% CI: 0.89–1.19, *p* = 0.700)) were attenuated to the null. This indicates that IL-6 activity, rather than generalized inflammation as a whole, could at least in part underly the associations of smoking with depression.

#### Assessment of bias and reliability of MR-Egger results

As MR-Egger tends to suffer from low statistical power and is particularly susceptible to bias from weak instruments^51^, we present assessment of reliability of the MR-Egger results. I^2^_GX_ represents the variants of the SNP-exposure associations. MR-Egger is only recommended when this is large (over 90%). I^2^_GX_ for the data used in the analyses with smoking as an exposure (Supplementary Table 5), suggested that the results from the MR-Egger analyses with smoking as an exposure may be strongly biased and are not reliable; therefore, in this case the IVW estimate is preferred^53,57^.

#### Steiger filtering

According to the Steiger filtering analyses, when lifetime smoking is used as the exposure, 125 out of 126 IVs were more strongly associated to lifetime smoking than to depression, suggesting that the IVs used do represent the causal effect of smoking on depression and do not support reverse causality. Similarly, when using depression as the exposure, 48 out of the 50 IVs assessed were more strongly associated with depression, suggesting that depression may lead to increased lifetime smoking.

*Additional analyses with an alternative CRP GWAS*^58^ (not UK Biobank based) are presented in Supplementary Table 6.

*Additional analyses taking into account correlation between instruments* are presented in Supplementary Tables 7 and 8.

## Discussion

### Summary of findings

In this study, we present comprehensive Mendelian randomization analyses testing the direction and potential causality of association between smoking, CRP, and depression. Our results suggest potentially causal bi-directional associations of smoking with depression and CRP levels. However, there was no evidence for a potentially causal association between CRP levels and depression, or for the effect of IL-6 activity on depression. We also found no evidence that inflammation-related smoking SNPs were associated with depression, and did not find an attenuation of the effect of smoking on risk of depression when adjusting for CRP in MVMR. However, we did find evidence for an attenuation of the effect of smoking on risk of depression when adjusting for IL-6 activity in MVMR. Together, these results suggest that while generalized inflammation may not be responsible for the associations of smoking and depression, the IL-6 pathway specifically may warrant further investigation as a potential biological mechanism underlying the effect of smoking on depression. The results obtained were consistent through the different sensitivity and robust analyses performed. Overall, the various robust methods performed relying on different assumptions, provide strong evidence to support the potential causal role of lifetime smoking on depression.

### Smoking and depression

For the relationship between smoking and depression, our results, using improved genetic instruments compared to prior studies, are consistent with previous evidence supporting a reciprocal association between these variables. Observational evidence has shown that depression can trigger smoking commencement and make its cessation more challenging^59–63^. Moreover, smoking has been shown to lead to depression, and smoking cessation has been associated with improved depressive outcomes and decreased depression symptomatology^63–65^. Wootton et al., using IVW methods and using data partially overlapping with that used in this study, obtained an OR of 1.99 (95% CI: 1.71–2.32; *p* < 0.001) for the causal role of lifetime smoking on depression, and an OR of 1.10 (95% CI : 1.02–1.17; *p* < 0.001) for the reciprocal relationship between depression and smoking^24^. The main differences between this analysis and that in Wootton et al. (2019) is that the Howard et al. (2018) depression GWAS that informed our study had a larger sample size and incorporated cases with a broader depression definition, including broad depression (help-seeking for problems with nerves, anxiety, tension, or depression) and probable major depressive disorder. This broader definition allows for an increase in in statistical power resulting from the larger number of cases encompassed may result in a loss of specificity^65^. Nevertheless, the results obtained are very similar to those from Wootton et al. (2019) (we found an odds ratio of 2.01 for effect of smoking on depression compared to Wootton et al. odds ratio of 1.99), suggesting that a despite the lack of a formal MDD diagnosis in all of the cases used, the broader depression definition used remains relevant for MR analysis^27,66^. Overall, Steiger filtering results support a bidirectional causal relationship between lifetime smoking and depression, likely through different pathways represented by the two IV sets, rather than simple genetic correlation.

The association between smoking and depression has important implications; it strengthens the case for primary prevention of smoking and stop-smoking initiatives, and raises the question, for future research, of whether smoking cessation initiatives are effective treatments for depression.

### Smoking and CRP

Evidence supporting a causal relationship between smoking and elevated CRP levels has been extensively documented in observational studies: with higher CRP levels in smokers^13–15,67–80^, and decreased CRP levels after smoking cessation^14,15,67,72,74–77,79,80^. This study provides new evidence to support a causal relationship between smoking behavior and CRP levels using Mendelian randomization techniques. Furthermore, this study supported a very small, but significant, effect (odds ratio of 1.03) of CRP levels on smoking lifetime index, which may be clinically negligible. Further study would be required to investigate which aspects of lifetime smoking CRP is causally associated with (as lifetime smoking is a composite measure reflecting both initiation and persistence of smoking).

### CRP and depression

Our results did not provide strong support for a potentially causal relationship between CRP and depression or vice versa, though several observational studies have demonstrated that patients with depression have significantly higher CRP levels when compared to those without depression^26,34,81–84^. Of previously published MR studies assessing this relationship^20,25,26^, our prior study (Khandaker et al. (2020)) found evidence for a potentially causal association between CRP levels and depression using data from the UK Biobank cohort. More recently, using the MR approach Kappelmann and colleagues have reported that inflammatory markers like CRP and IL-6 are associated with specific symptoms of depression, such as suicidality^85^. Using symptom-level data from the UK Biobank and Dutch NESDA cohorts we have reported observational and MR associations for CRP and IL-6 with somatic/neurovegetative symptoms of depression such as fatigue and sleeping difficulties^86^. Taken together, current evidence from epidemiological and genetic MR studies is consistent with inflammation being potentially causally related to certain symptoms of depression, namely somatic/neurovegetative symptoms, though MR evidence for a potentially causal role of CRP on the syndrome of depression as outcome is mixed. We note that there are commonly observed phenotypic associations between CRP and heart disease, but mendelian randomisation studies do not indicate those are causal^52^. On the question of whether depression causes raised CRP, we do note that whilst there was no association between genetically predicted depression and CRP as assessed with the IVW method, the more robust methods of MR-PRESSO and contamination mixture did indicate such an effect; these conflicting results make it hard to draw definitive conclusions on whether depression causes raised CRP.

Overall, these results do not support a role for CRP-indexed inflammation in the development of depression. This null effect contrasts with a prior finding, using the same CRP GWAS and a similar methodology, that serum CRP is causally associated with another multifactorial etiology phenotype—namely age-related macular degeneration—indicating that the CRP genetic instruments we used are capable of revealing positive causal relationships between serum CRP and disease. Randomized control trials have shown that using of anti-inflammatory agents, such as celecoxib or infliximab, can successfully improve depression outcomes in patients with elevated CRP levels and patients with treatment-resistant depression^87^, strongly suggesting that inflammation has a crucial role in the pathogenesis of at least certain types of depression^58,87^. If inflammation is only causally relevant in *certain subtypes* of depression with their own distinct etiology and pathogenesis, our approach would not necessarily detect this. However, our results remain consistent with the possibility that inflammation may cause depression, considered as a unitary entity, via *non-CRP mediated* processes. Our research strongly suggests that future studies examining the effect of inflammatory cytokines on depression should broaden their scope beyond CRP.

### Inflammation as a mediator

The lack of difference in results of the analyses using inflammatory-related and non-inflammatory related smoking IVs, suggest that there is no clear distinct CRP mediated inflammatory causal pathway mediating the causal relationship detected between smoking and depression. Furthermore, MVMR analyses showed that adjusting the effect of smoking on depression for CRP levels did not result in a significant estimate for the effect of CRP levels. However, MVMR analysis did show that adjusting the effect of smoking for IL-6 activity did significantly attenuate the estimate effect of smoking on depression, which is consistent with the hypothesis that smoking’s effects on depression are mediated through IL-6 activity; this hypothesis therefore merits further investigation.

### Limitations

Lifetime smoking index and depression are both behavioral variables, with complex etiologies and hard to measure phenotypes. Furthermore, the statistical approach to IV selection use increases the risk of bias from horizontal pleiotropy^88^. A genetic variant may present horizontal pleiotropy, meaning that it is not only associated with the exposure, but it is additionally associated with one or more risk factors for the outcome, biasing the true association between the exposure and outcome^89^. However, the robust methods used, including MR-Egger, MR-PRESSO, and MR contamination mixture, allow MR analyses to be conducted in the presence of horizontal pleiotropy. The various sensitivity analyses we performed generally provide supporting evidence for the robustness of the findings.

It is important to note that the smoking and CRP data were only from the UK Biobank, as in a one-sample MR study. One-sample MR IVW analyses using weak instruments, as assessed by the F-statistic, could lead to an overestimation of the causal effect between the exposure and the outcome assessed^90,91^. Nevertheless, previous studies performed in non-overlapping samples have demonstrated the same direction, with a very similar effect size, of the effect between smoking and depression^24^. Furthermore, we used a smaller (47 independent IVs), non-overlapping CRP GWAS^37^, which showed a similar reciprocal associations between smoking and CRP, and similar CRP and depression findings (Supplementary Table 6).

## Conclusion

The results from this study add on to the growing body of evidence supporting the bidirectionality of the causal relationship between smoking and depression. Furthermore, this study strengthens the evidence for a causal role of smoking on CRP levels. However, we do not find evidence for a potentially causal role for CRP on depression or for potentially mediating role for CRP on the association between smoking and depression, though we did find evidence for a potentially mediating role for IL-6 activity on this association. Further research is needed to understand potential mechanisms for bidirectional association between smoking and depression and to develop effective interventions.

## Supplementary Information

Supplementary Information.
