# Supplementary material for: Applying Mendelian randomization to appraise causality in relationships between smoking, depression and inflammation
Source: Sci Rep. 2022 Sep 3;12:15041. doi: 10.1038/s41598-022-19214-4 (PMC9440889; doi:10.1038/s41598-022-19214-4)
Supplement: Supplementary file 1 — Supplementary Information. [file 41598_2022_19214_MOESM1_ESM.docx]

**Supplementary Information**

**Applying Mendelian randomization to appraise causality in relationships between smoking, depression and inflammation**

Galan, D.^1*^, Perry, B.I. ^2,3*^, Warrier, V. ^2,3^, Davidson, C.C. ^2^, Stupart, O. ^2^, Easton, D. ^1^, Khandaker, G.M. ^2,3,4,5,6^, Murray, G.K. ^2,3,7^

^1^Department of Public Health and Primary Care, University of Cambridge, Cambridge, UK

^2^Department of Psychiatry, University of Cambridge, Cambridge, UK

^3^Cambridgeshire and Peterborough NHS Foundation Trust, Cambridge, UK

^4^MRC Integrative Epidemiology Unit, Population Health Sciences, Bristol Medical School, University of Bristol, Bristol, UK

^5^Centre for Academic Mental Health, Population Health Sciences, Bristol Medical School, University of Bristol, Bristol, UK

^6^Avon and Wiltshire Mental Health Partnership NHS Trust, Bristol, UK

^7^Program in Complex Trait Genomics, Institute of Molecular Bioscience, University of Queensland, Brisbane, Australia

*Equal contributions

*Supplemental Table 1. Depression case definitions used in the different cohorts combined for the depression*

*GWAS summary statistics from the PGC^27,29^.*

| Study | Cohort | Depression Definition |
| --- | --- | --- |
| Howard et al.  (2018) | UK Biobank | (1) Self-reported past help-seeking for problems with “nerves, anxiety, tension or depression”, (2) self-reported depressive symptoms with associated impairment, and (3) MDD identified from ICD-9 or ICD-10-coded hospital admission records. |
| Wray et al. (2018) | PGC29^*^ | Structured diagnostic interviews. |
|  | deCODE | Electronic medical records. |
|  | GenScotland | Structured diagnostic interviews. |
|  | GERA | Electronic medical records. |
|  | iPSYCH | Electronic medical records. |
|  | UK Biobank^+^ | (1) Self-reported MDD symptoms, (2) self-reported  MDD treatment, or (3) electronic medical records. |
|  | 23andMe^&^ | Self-reported diagnosis or treatment for clinical depression by a medical professional |

*^*^ PGC29 includes 29 different cohorts^30^.*

*^+^ UK Biobank samples from Wray et al were excluded to prevent overlapping data with Howard et al. (2018) that included UK Biobank data. ^&^ 23andMe samples are not publicly available and therefore were not included.*

*Supplementary Table 2. Instrumental variable selection using statistical methods.*

| Variable | GWAS SNPs | Significant SNPs | Independents SNPs | Variance Explained (R^2^) | F-statistic^1^ |
| --- | --- | --- | --- | --- | --- |
| Lifetime Smoking | 7,683,352 | 10,415 | 126 | 0.0036 | 13.26 |
| CRP | 8,927,092 | 60,177 | 526 | 0.11 | 118.78 |
| Depression | 8,484,301 | 4,625 | 50 | 0.023 | 193.74 |

^1^ F-statistic values were estimated using previously reported R^2^ (variance of the variable explained by the SNPs used in the original analysis) and may not represent the true value for the analyses included here.

*
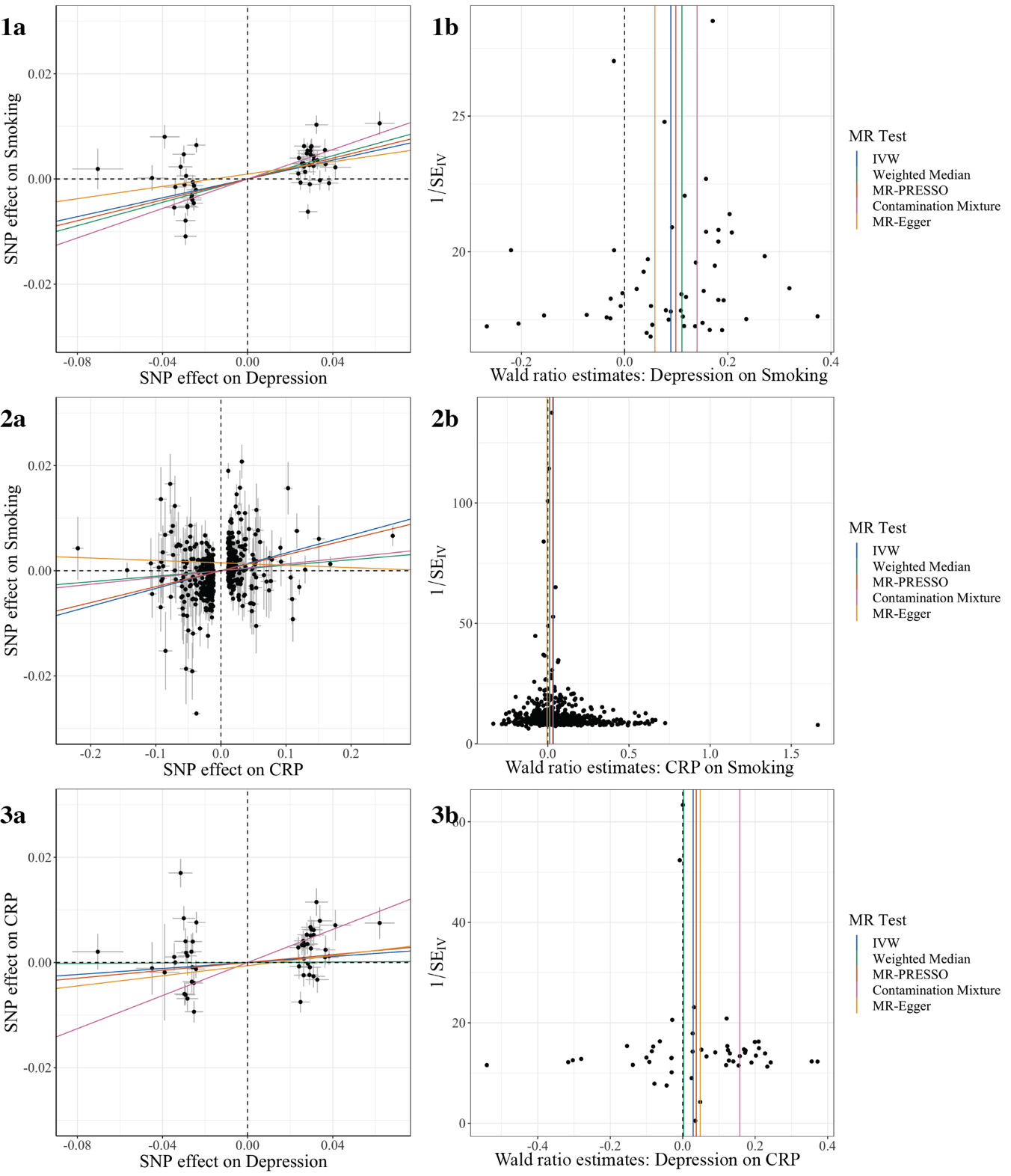
*

Supplemental Figure 1. Reciprocal analyses: Effect of (1) Depression vs Smoking, (2) CRP vs Smoking, (3) Depression vs CRP. (a) Scatterplot showing the relationship between the variant-depression associations (x-axis) and the variant-smoking associations (y-axis) with standard error bars. The slopes of the colored lines correspond to the estimated causal effect obtained with each method used. (b) Funnel plot showing the relationship between the causal effect of the exposure on the outcome estimated using the Wald ratio estimate for each IV (x-axis) against the inverse of the standard error of the such estimate (y-axis). Vertical lines show the causal estimates using all SNPs combined into a single instrument for each of five different methods.

*
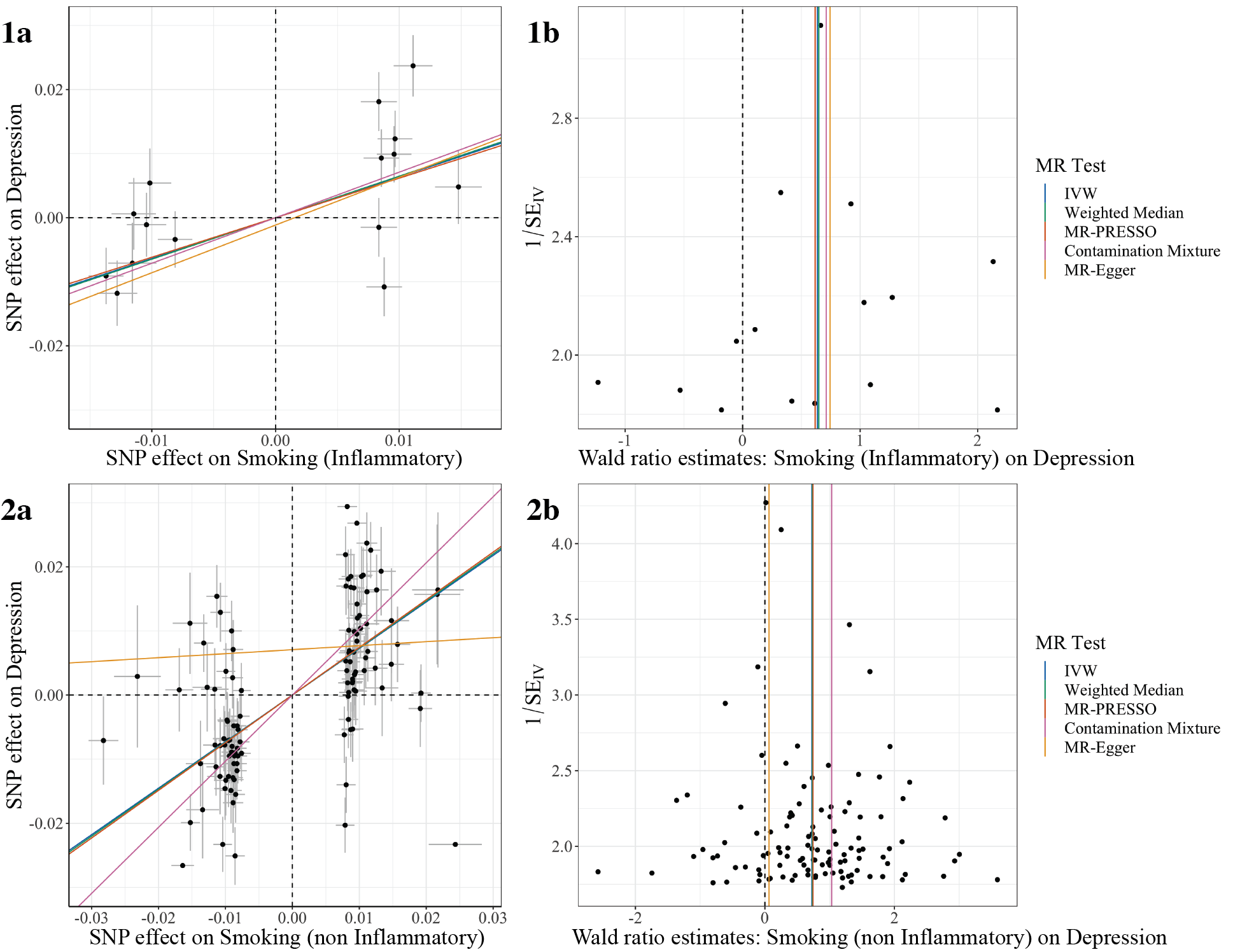
*

Supplemental Figure 2. Inflammatory analyses: Effect of (1) smoking (inflammatory) vs depression, and (2) smoking (non inflammatory) vs depression. (a) Scatterplot showing the relationship between the variant-depression associations (x-axis) and the variant-smoking associations (y-axis) with standard error bars. The slopes of the colored lines correspond to the estimated causal effect obtained with each method used. (b) Funnel plot showing the relationship between the causal effect of the exposure on the outcome estimated using the Wald ratio estimate for each IV (x-axis) against the inverse of the standard error of the such estimate (y-axis). Vertical lines show the causal estimates using all SNPs combined into a single instrument for each of five different methods.


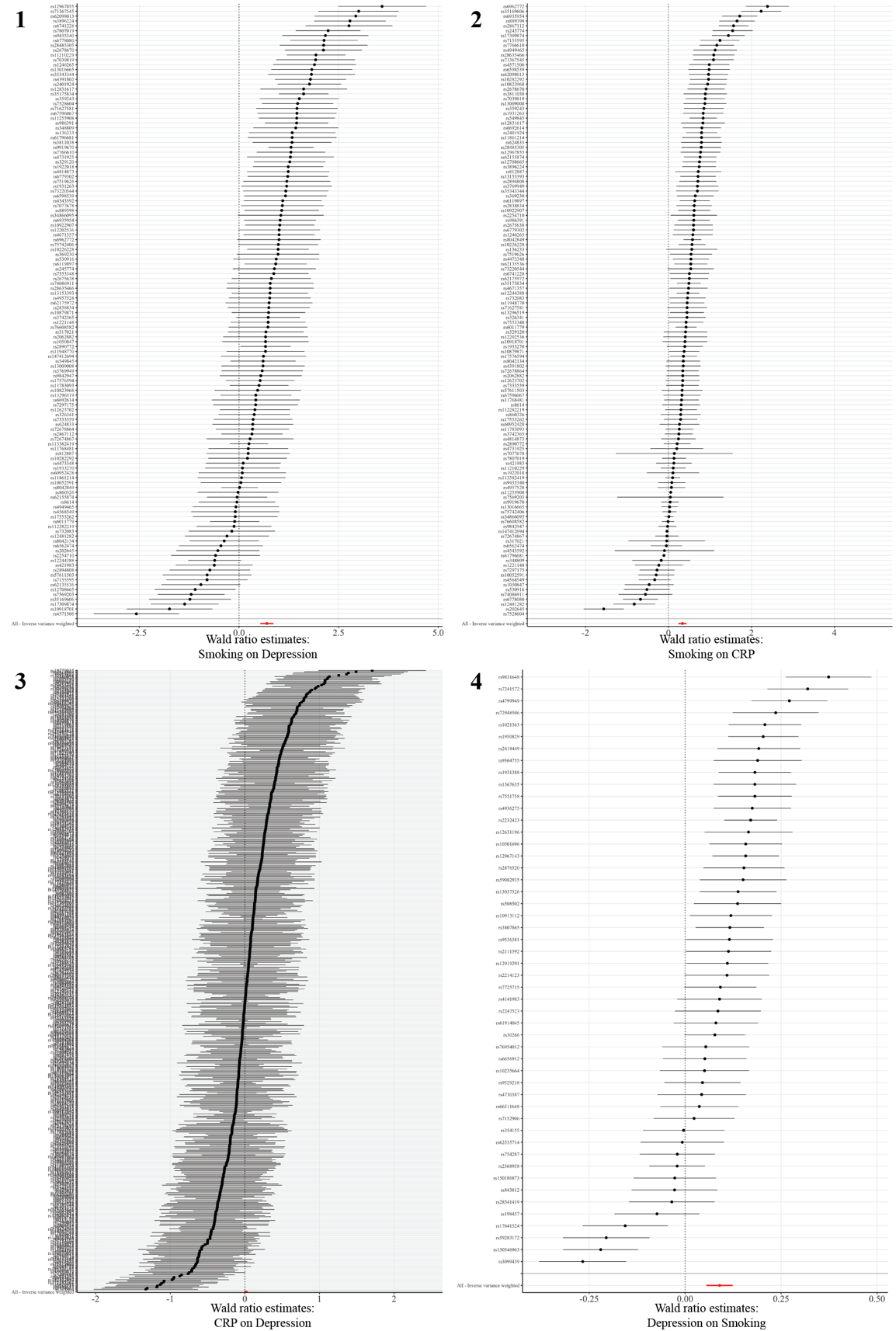
Supplemental Figure 3. Forest plots showing the Wald ratio estimates for each IV with 95% confidence interval error bars, and comparing against the causal effect as estimated using the IWV method (in red) for the effect of (1) smoking on depression, (2) smoking on CRP, (3) CRP on depression, (4) depression on smoking, (5) CRP on smoking, (6) depression on CRP, (7) smoking (inflammatory) on depression, and (8) smoking (non inflammatory) on depression.

*
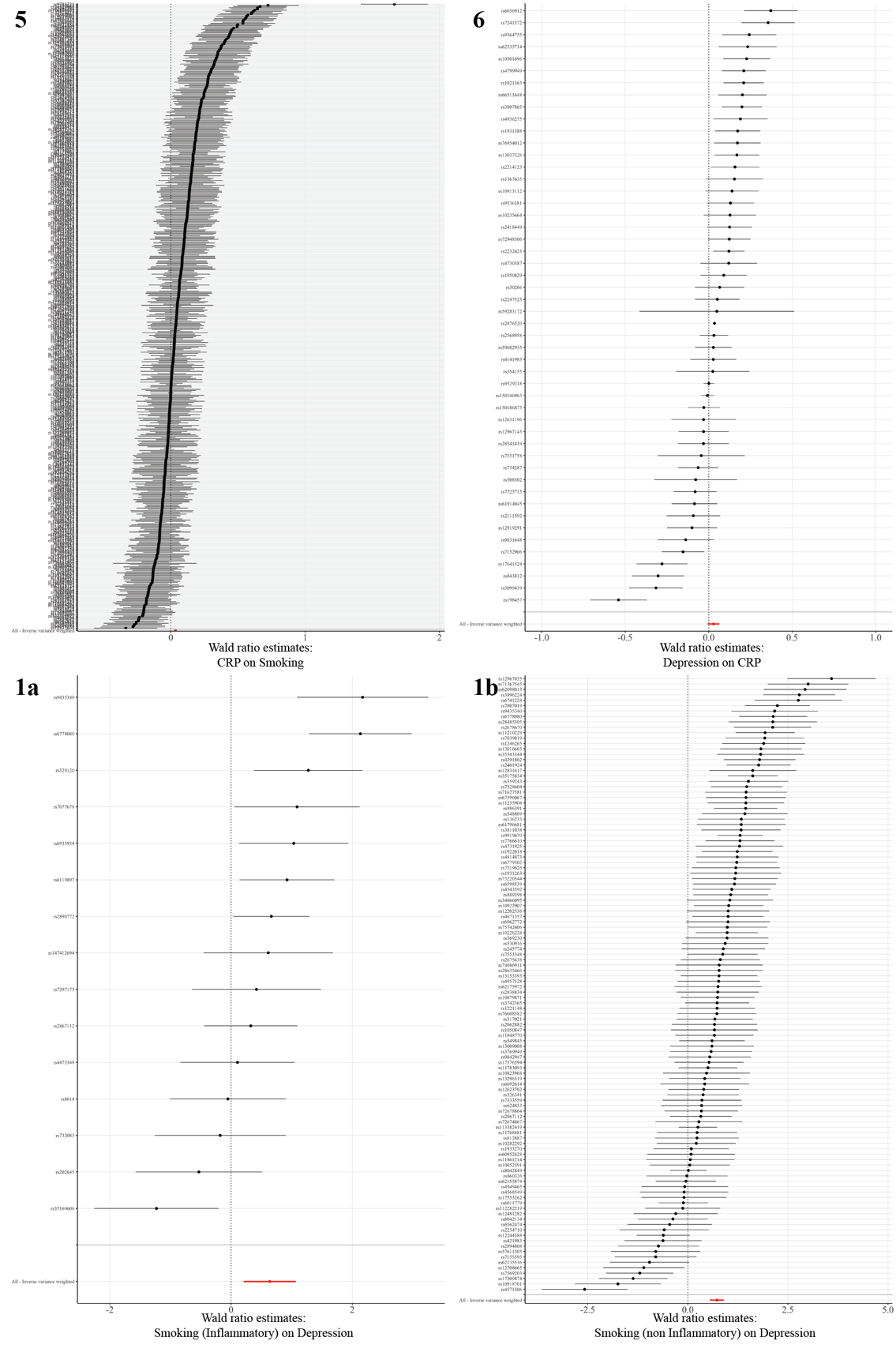
Supplemental Figure 3 (Continued)*

*
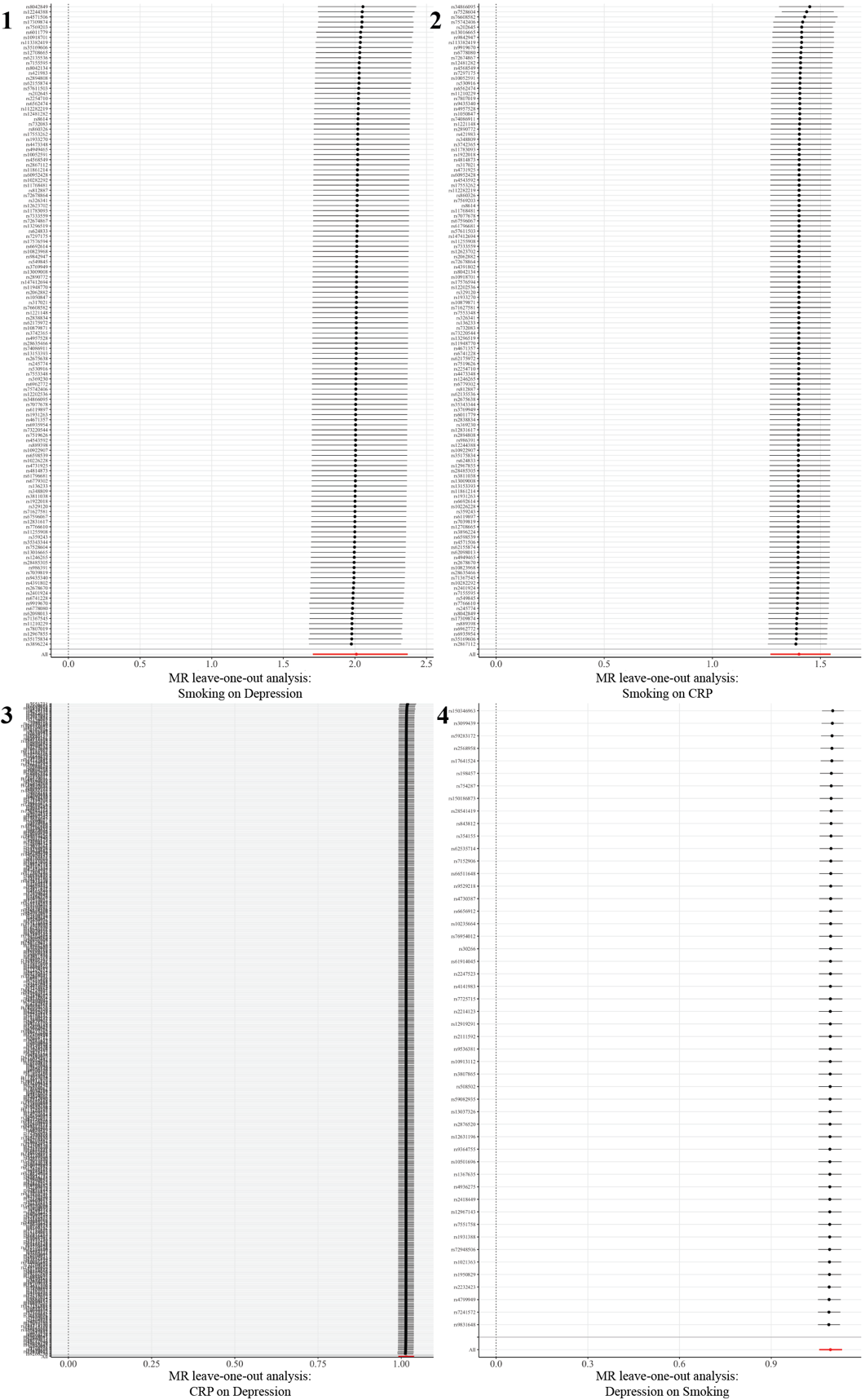
* Supplemental Figure 4. Leave-one-out analysis plot showing the IVW estimates with 95% confidence interval error bars for all of the IVs, excluding the one listed in the y-axis, for (1) smoking on depression, (2) smoking on CRP, (3) CRP on depression, (4) depression on smoking, (5) CRP on smoking, (6) depression on CRP, (7) smoking (inflammatory) on depression, and (8) smoking (non inflammatory) on depression.

*
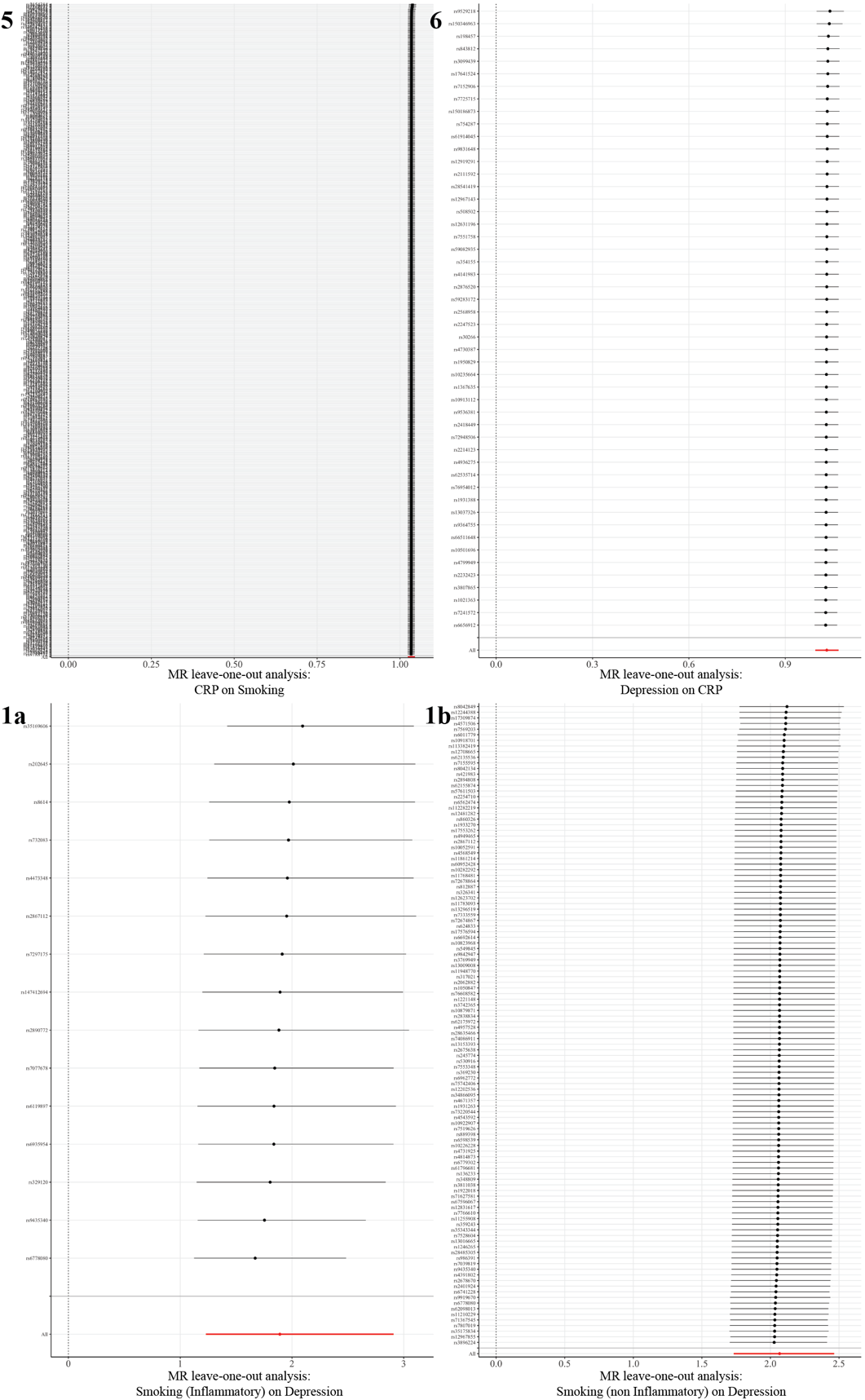
Supplemental Figure 4 (Continued)*

*Supplementary Table 3. Sensitivity analyses for the univariable MR analyses.*

| Exposure | Outcome | Method | N_SNPs_ | OR | SE | Low CI | Up CI | p-val |
| --- | --- | --- | --- | --- | --- | --- | --- | --- |
| Smoking | Depression | IVW | 126 | 2.01 | 0.08 | 1.70 | 2.36 | 7.78E-17 |
|  |  | Weighted Median | 126 | 2.07 | 0.08 | 1.79 | 2.41 | 1.07E-21 |
|  |  | MR-PRESSO | 116 | 2.05 | 0.07 | 1.79 | 2.34 | 3.70E-18 |
|  |  | Contamination | 79 | 2.51 | NA | 2.14 | 3.13 | 1.90E-17 |
|  |  | MR-EGGER | 126 | 1.12 | 0.33 | 0.59 | 2.14 | 0.727 |
| Smoking | CRP | IVW | 126 | 1.40 | 0.05 | 1.27 | 1.55 | 2.89E-11 |
|  |  | Weighted Median | 126 | 1.38 | 0.04 | 1.28 | 1.48 | 7.49E-18 |
|  |  | MR-PRESSO | 111 | 1.45 | 0.03 | 1.36 | 1.54 | 2.49E-21 |
|  |  | Contamination | 87 | 1.72 | NA | 1.63 | 1.80 | 1.32E-24 |
|  |  | MR-EGGER | 126 | 1.39 | 0.19 | 0.96 | 2.01 | 0.080 |
| CRP | Depression | IVW | 512 | 1.01 | 0.01 | 0.99 | 1.04 | 2.25E-01 |
|  |  | Weighted Median | 512 | 1.00 | 0.02 | 0.97 | 1.03 | 8.38E-01 |
|  |  | MR-PRESSO | 504 | 1.01 | 0.01 | 0.99 | 1.03 | 2.76E-01 |
|  |  | Contamination | 380 | 1.00 | NA | 0.98 | 1.01 | 1.000 |
|  |  | MR-EGGER | 512 | 0.98 | 0.02 | 0.95 | 1.01 | 0.272 |
| IL-6 effect on CRP levels | Depression | IVW | 7 | 0.93 | 0.05 | 0.85 | 1.02 | 0.126 |
|  |  | Weighted Median | 7 | 0.96 | 0.04 | 0.88 | 1.04 | 0.308 |
|  |  | MR-PRESSO | 7 | 0.96 | 0.04 | 0.88 | 1.04 | 0.308 |
|  |  | Contamination | 7 | 0.95 | NA | 0.87 | 1.04 | 0.446 |
|  |  | MR-EGGER | 7 | 0.99 | 0.16 | 0.73 | 1.35 | 0.963 |
| Depression | Smoking | IVW | 50 | 1.09 | 0.02 | 1.06 | 1.13 | 3.15E-07 |
|  |  | Weighted Median | 50 | 1.12 | 0.01 | 1.08 | 1.15 | 2.02E-16 |
|  |  | MR-PRESSO | 43 | 1.10 | 0.01 | 1.08 | 1.13 | 3.03E-10 |
|  |  | Contamination | 31 | 1.15 | NA | 1.13 | 1.19 | 4.64E-10 |
|  |  | MR-EGGER | 50 | 1.06 | 0.09 | 0.89 | 1.27 | 0.529 |
| CRP | Smoking | IVW | 521 | 1.03 | 0.01 | 1.02 | 1.04 | 1.71E-10 |
|  |  | Weighted Median | 521 | 1.01 | 0.01 | 1.00 | 1.02 | 0.050 |
|  |  | MR-PRESSO | 493 | 1.03 | 0.00 | 1.02 | 1.04 | 3.43E-11 |
|  |  | Contamination | 349 | 1.01 | NA | 1.01 | 1.02 | 1.08E-03 |
|  |  | MR-EGGER | 521 | 1.00 | 0.01 | 0.98 | 1.01 | 0.533 |
| IL-6 effect on CRP levels | Smoking | IVW | 7 | 1.06 | 0.02 | 1.03 | 1.09 | <0.001 |
|  |  | Weighted Median | 7 | 1.05 | 0.01 | 1.02 | 1.08 | <0.001 |
|  |  | MR-PRESSO | 7 | 1.06 | 0.02 | 1.03 | 1.09 | <0.001 |
|  |  | Contamination | 7 | 1.04 | NA | 1.03 | 1.08 | <0.001 |
|  |  | MR-EGGER | 7 | 1.01 | 0.05 | 0.91 | 1.12 | 0.900 |
| Depression | CRP | IVW | 50 | 1.03 | 0.02 | 0.99 | 1.06 | 0.110 |
|  |  | Weighted Median | 50 | 1.00 | 0.01 | 0.98 | 1.03 | 0.856 |
|  |  | MR-PRESSO | 44 | 1.04 | 0.01 | 1.01 | 1.06 | 0.009 |
|  |  | Contamination | 26 | 1.17 | NA | 1.14 | 1.20 | 0.001 |
|  |  | MR-EGGER | 50 | 1.05 | 0.10 | 0.85 | 1.28 | 0.642 |
| Smoking (Inflammatory) | Depression | IVW | 15 | 1.89 | 0.22 | 1.23 | 2.92 | 0.004 |
|  |  | Weighted Median | 15 | 1.91 | 0.19 | 1.32 | 2.77 | 0.001 |
|  |  | MR-PRESSO | 13 | 1.85 | 0.18 | 1.31 | 2.61 | 0.004 |
|  |  | Contamination | 9 | 2.04 | NA | 1.34 | 3.10 | 0.012 |
|  |  | MR-EGGER | 15 | 2.11 | 1.22 | 0.19 | 22.87 | 0.540 |
| Smoking (non-Inflammatory) | Depression | IVW | 114 | 2.07 | 0.09 | 1.73 | 2.46 | 6.97E-16 |
|  |  | Weighted Median | 114 | 2.08 | 0.08 | 1.79 | 2.44 | 5.93E-20 |
|  |  | MR-PRESSO | 103 | 2.07 | 0.07 | 1.80 | 2.39 | 6.59E-18 |
|  |  | Contamination | 72 | 2.80 | NA | 2.14 | 3.56 | 4.99E-16 |
|  |  | MR-EGGER | 114 | 1.06 | 0.34 | 0.54 | 2.10 | 0.857 |

Abbreviations: “Smoking (Inflammatory)” indicates smoking IVs previously associated with inflammation. Smoking (non-Inflammatory) indicates smoking IVs not previously associated with inflammation. “Low CI” lower limit of 95% confidence interval. “Up CI” upper limit of 95% confidence interval.

*Supplementary Table 4. MVMR Estimates. It shows the overall estimates of lifetime smoking on depression when adjusted by either the mediators (CRP or IL-6 effect on CRP), as well as the effect of the mediator itself.*

Abbreviations: nSNP number of SNPs. IL-6: IL-6 effect on CRP

| **MVMR Analysis** | **nSNPs** | **Exposures** | **Outcome** | **OR** | **Low CI** | **Up CI** | **pval** |
| --- | --- | --- | --- | --- | --- | --- | --- |
| CRP mediation of Smoking- Depression | 169 | CRP | Depression | 0.98 | 0.92 | 1.04 | 0.48 |
|  |  | Smoking | Depression | 2.00 | 1.62 | 2.46 | 7.35E-11 |
| IL-6 mediation of Smoking-Depression | 125 | IL-6 activity | Depression | 1.03 | 0.89 | 1.19 | 0.700 |
|  |  | Smoking | Depression | 0.94 | 0.76 | 1.17 | 0.602 |

Abbreviations: “Low CI” lower limit of 95% confidence interval. “Up CI” upper limit of 95% confidence interval.

*Supplementary Table 5. MR-Egger analyses - calculated I^2^_GX_ and Q_R_ values.*

| Exposure | Outcome | SNPs | *I^2^_GX_* | Q_R_ |
| --- | --- | --- | --- | --- |
| Smoking | Depression | 126 | 0.64 | 0.974 |
| Smoking | CRP | 126 | 0.64 | 1 |
| CRP | Depression | 512 | 0.98 | 0.986 |

*Supplementary Table 6. Robust MR Estimates for the univariable MR analyses using a non-UK Biobank CRP GWAS.*

| Exposure | Outcome | Method | N_SNPs_ | OR | Low CI | Up CI | p-val |
| --- | --- | --- | --- | --- | --- | --- | --- |
| Smoking | CRP | IVW | 125 | 1.35 | 1.19 | 1.53 | <0.001 |
| Smoking | CRP | Weighted Median | 125 | 1.31 | 1.15 | 1.50 | <0.001 |
| Smoking | CRP | MR-PRESSO | 121 | 1.34 | 1.21 | 1.49 | <0.001 |
| Smoking | CRP | Contamination | 81 | 1.83 | 1.51 | 2.06 | <0.001 |
| Smoking | CRP | MR-EGGER | 125 | 1.13 | 0.66 | 1.94 | 0.653 |
| CRP_trans | Depression | IVW | 46 | 1.00 | 0.97 | 1.03 | 0.967 |
| CRP_trans | Depression | Weighted Median | 46 | 0.99 | 0.96 | 1.02 | 0.561 |
| CRP_trans | Depression | MR-PRESSO | 44 | 1.00 | 0.97 | 1.03 | 0.938 |
| CRP_trans | Depression | Contamination | 35 | 0.99 | 0.97 | 1.01 | 0.641 |
| CRP_trans | Depression | MR-EGGER | 46 | 1.00 | 0.95 | 1.05 | 0.906 |
| CRP_trans | Smoking | IVW | 46 | 1.01 | 1.00 | 1.03 | 0.075 |
| CRP_trans | Smoking | Weighted Median | 46 | 1.01 | 0.99 | 1.02 | 0.445 |
| CRP_trans | Smoking | MR-PRESSO | 44 | 1.01 | 1.00 | 1.02 | 0.019 |
| CRP_trans | Smoking | Contamination | 34 | 1.01 | 1.00 | 1.03 | 0.123 |
| CRP_trans | Smoking | MR-EGGER | 46 | 1.01 | 0.99 | 1.04 | 0.285 |
| Depression | CRP | IVW | 50 | 1.00 | 0.93 | 1.07 | 0.959 |
| Depression | CRP | Weighted Median | 50 | 1.02 | 0.95 | 1.09 | 0.636 |
| Depression | CRP | MR-PRESSO | 48 | 1.02 | 0.96 | 1.08 | 0.548 |
| Depression | CRP | Contamination | 30 | 1.11 | 0.86 | 1.23 | 0.209 |
| Depression | CRP | MR-EGGER | 50 | 0.88 | 0.61 | 1.27 | 0.499 |

Abbreviations: “Low CI” lower limit of 95% confidence interval. “Up CI” upper limit of 95% confidence interval.

*Supplementary Table 7. Correlation adjusted analysis*

| **Exposure** | **Outcome** | **N SNPs** | **Correlation-Adjusted Univariable IVW Analysis** | | | |
| --- | --- | --- | --- | --- | --- | --- |
|  |  |  | **OR** | **Low CI** | **Up CI** | **p-val** |
| Smoking (all) | Depression | 126 | 1.98 | 1.69 | 2.31 | <0.001 |
| Smoking (inf) | Depression | 15 | 1.93 | 1.21 | 3.07 | 0.006 |
| CRP | Depression | 229 | 0.99 | 0.97 | 1.03 | 0.903 |
| CRP | Smoking | 41 | 1.02 | 1.01 | 1.03 | <0.001 |
| Smoking (all) | CRP | 126 | 1.40 | 1.27 | 1.54 | <0.001 |
| IL-6 Activity | Depression | 7 | 0.95 | 0.86 | 1.06 | 0.395 |
| IL-6 Activity | Smoking | 7 | 1.05 | 1.00 | 1.10 | 0.050 |

Abbreviations: “Low CI” lower limit of 95% confidence interval. “Up CI” upper limit of 95% confidence interval.

*Supplementary Table 8. Multivariable analysis with correlation adjustment*

| **MVMR Analysis** | **N SNPs** | **Exposures** | **Outcome** | **Multivariable IVW Analysis** | | | |
| --- | --- | --- | --- | --- | --- | --- | --- |
|  |  |  |  | **OR** | **Low CI** | **Up CI** | **pval** |
| CRP mediation of Smoking- Depression | 170 | CRP | Depression | 1.02 | 0.96 | 1.09 | 0.49 |
|  |  | Smoking | Depression | 2.02 | 1.65 | 2.48 | 1.16 E-11 |
| IL-6 mediation of Smoking-Depression | 125 | IL-6 activity | Depression | 1.01 | 0.88 | 1.17 | 0.874 |
|  |  | Smoking | Depression | 0.92 | 0.74 | 1.14 | 0.427 |

Abbreviations: “Low CI” lower limit of 95% confidence interval. “Up CI” upper limit of 95% confidence interval.
